# Synthesis, Characterization and In Vitro Study of Synthetic and Bovine-Derived Hydroxyapatite Ceramics: A Comparison

**DOI:** 10.3390/ma11030333

**Published:** 2018-02-25

**Authors:** July Andrea Rincón-López, Jennifer Andrea Hermann-Muñoz, Astrid Lorena Giraldo-Betancur, Andrea De Vizcaya-Ruiz, Juan Manuel Alvarado-Orozco, Juan Muñoz-Saldaña

**Affiliations:** 1 Centro de Investigación y de Estudios Avanzados del IPN, Unidad Querétaro, Libramiento Norponiente #2000, Fraccionamiento Real de Juriquilla, Santiago de Querétaro 76230, Mexico; jrincon@cinvestav.mx (J.A.R.-L.); jhermann@cinvestav.mx (J.A.H.-M.); agiraldo@cinvestav.mx (A.L.G.-B.); 2 Departamento de Toxicología, Centro de Investigación y de Estudios Avanzados del IPN, Unidad Zacatenco, Av. Instituto Politécnico Nacional 2508, Col. San Pedro Zacatenco, Delegación Gustavo A. Madero, Ciudad de Mexico 07360, Mexico; avizcaya@cinvestav.mx; 3 Centro de Ingeniería y Desarrollo Industrial, Av. Playa Pie de la Cuesta No. 702, Desarrollo San Pablo, Santiago de Querétaro 76125, Mexico; juan.alvarado@cidesi.edu.mx

**Keywords:** Bioceramics, bovine-derived hydroxyapatite, sintering, biocompatibility

## Abstract

The physicochemical properties and biological behavior of sintered-bovine-derived hydroxyapatite (BHAp) are here reported and compared to commercial synthetic-HAp (CHAp). Dense ceramics were sintered for 2 h and 4 h at 1200 °C to investigate their microstructure–structure–in-vitro behavior relationship for both HAp ceramics. Densification was directly proportional to sintering time, showing a grain coarsening behavior with a greater effect on BHAp. Lattice parameters, crystallite size, cell volume and *Ca*/*P* ratio were determined by Rietveld refinement of X-ray diffraction (XRD) patterns using GSAS^®^. Ionic substitutions (Na^+^, Mg^2+^, CO_3_^2−^) related to BHAp structure were associated with their position changes in the vibrational modes and correlated with the structural parameters obtained from the XRD analysis. Variations in the structural parameters and surface morphology were also evaluated after different soaking periods in simulated body fluid, which is associated with the formation of bone-like apatite layer and thus bioactivity. Mitochondrial activity (MTS) and lactate dehydrogenase (LDH) assays showed that the material released by the ceramics does not induce toxicity after exposure in human fetal osteoblastic (hFOB) cells. Furthermore, no statistically significant differences were found between the HAp obtained from different sources. These results show that BHAp can be used with no restrictions for the same biomedical applications as CHAp.

## 1. Introduction

A variety of materials based on calcium phosphate (CP) ceramics have been investigated as a clinical treatment for musculoskeletal system diseases [1,2]. For the last 25 years, hydroxyapatite (HAp) is by far the most studied within this group of CP materials, mainly for its ability to promote bone ingrowth due to its osteoconductive properties, thus reducing the risk of a local or systemic toxicity [2]. The HAp shows an adequate functional bioactive behavior in which the interaction with the physiological medium promotes the formation of a layer with the composition and structure of bone apatite on its surface, hereafter defined as bone-like apatite, accelerating the bonding between the tissue and the implant [3,4]. All the mentioned properties have driven the use of HAp for several biomedical applications such as bone replacement, tissue engineering, drug delivery, dental materials, bioactive coatings on metallic implants, among others [5,6,7].

Synthetic stoichiometric HAp, Ca_10_(PO_4_**)**_6_(OH)_2_ having typically a *Ca*/*P* ratio of 1.67 can exhibit either monoclinic or hexagonal crystal structures [8,9]. The hexagonal phase with the *P6*_3_/*m* space group, lattice parameters *a = b =* 9.432 Å, *c* = 6.881 Å is the most frequently reported and consist of unconnected, PO_4_^3−^ tetrahedra with Ca^2+^ in the interstitial space and a chain of OH^−^ ions along the c-axis to balance the unit cell charges [8,9,10]. In particular, biological apatites are substituted either by Na^+^, Mg^2+^, and K^+^ cations or F^−^, Cl^−^, SiO_4_^4−^ and CO_3_^2−^ anions, or in some cases by both [9,11,12,13]. Specifically, the occupancy of carbonate ions is either in the hydroxyl and the phosphate ions sites in the apatite structure, leading to an A- or B-type carbonated hydroxyapatites, respectively. If these substitutions take place simultaneously, an AB-type substitution occurs, as in the case of the bone mineral [8,9,14]. All these trace elements play a crucial role in the performance of hard tissue. Therefore, alternative methods to modify the HAp structure incorporating different ions seeking to improve the osteoconductive properties of synthetic HAp is of scientific interest [13].

HAp powder can be synthesized through different chemical methods or obtained from several processing routes and sources. Thus, efficient and low-cost methods to obtain HAp from natural sources are gaining in interest in the last years. In fact, it has been argued that HAp obtained from biowaste such as eggshells, bovine bones, fish-scales and fish bones leads to general properties and behavior comparable or even better than synthetic ones due to the similarities to bone apatites [13,15,16]. Thus, HAp extracted from biowaste represents an economic and environmentally viable route having also high scientific and technological interest [15].

In this study, the physicochemical properties of in-house prepared bovine-derived hydroxyapatite are evaluated and compared with those from a synthetic commercial origin, hereafter referred as BHAp and CHAp, respectively. The research is focused on structural aspects and their effect on its biological behavior seeking to demonstrate the potential of this biogenic HAp to be used in biomedical applications.

## 2. Material and Methods

### 2.1. Sample Preparation

BHAp was obtained by processing cortical bovine bones and prepared following a modified processing route described elsewhere in order to completely remove the organic material [17]. Bovine bones (2 years old) were acquired through certified local slaughterhouse. Briefly, the bovine bone was initially subjected to a pre-cleaning step to eliminate body fluids and remnant tissue adhered to the bone. Thereafter, bones were crushed and sent to a fat removal step process using solvents, followed by a milling process before sending the powder to final calcination step to assure the elimination of the organic material. CHAp powder (Himed^®^, Old Bethpage, NY, USA) was used as a reference material for comparative purposes. Both, CHAp and BHAp powders were sieved to obtain a particle size lower than 75 μm. Round shaped green samples were uniaxially pressed at 35 kg/cm^2^ using a die with 10 mm in diameter and 0.25 g per sample. The green samples were sintered at 1200 °C for 2 h and 4 h with a heating rate of 5 °C/min and cooling rate of 10 °C/min in a chamber furnace (Thermolyne 46100, Thermofisher Scientific, Waltham, MA, USA).

### 2.2. Sample Characterization

#### 2.2.1. Powder Size Distribution, Chemical Composition and Microstructure of Ceramics

Particle size distribution of both types of HAp was measured at least five times using a laser diffractometer (HELOS/BR, Sympatec GmbH, Clausthal-Zellerfeld, Germany). The measurements were performed using a RODOS technique for dry powder, where samples were placed in the powder feeder and air pressurized at 0.2 bar.

The Na, Mg, Ca, P and heavy metals contents from both HAp powders were obtained by Inductively Coupled Plasma-Optical emission spectroscopy, ICP-OES (Ultima2, Horiba, NY, USA). Characterization of powder morphology and microstructure of sintered ceramics, before and after SBF exposure was performed by scanning electron microscopy- electron microprobe analyzer, SEM-EPMA (JXA-8530F, JEOL, Tokyo, Japan) at 6 kV electron acceleration voltage and a secondary electron detector. Grain size was quantified following the ASTM E112 standard [18] (lineal intercept method) from at least four micrographs recorded at different magnifications (500–2500×).

#### 2.2.2. Mechanical Properties

Microhardness of the HAp ceramics was measured on polished samples with a mirror finish using a Vickers hardness tester (THV-1D, KAIRDA group company, Beijing, China) with a 300 g load and 10 s dwell time. An average value was taken from five indents on two samples of each HAp type.

#### 2.2.3. Structural Characteristics

Structural characterization was performed by X-ray diffraction, XRD (DMax 2100, Rigaku, TX, USA) with a monochromatic CuKα radiation (*λ* = 1.5406 Å) operating at 30 kV and 20 mA. The XRD patterns were recorded between 20 to 70° on a 2θ scale in steps of 0.02° intervals with a counting time of 0.6 s at each step. The powder and sintered specimens were measured at a fixed angle of 5°. The structural changes after different immersion times in simulated body fluid (SBF) were measured on the ceramics surface, at a grazing incidence angle of 1°. In order to characterize and quantify the phase fraction in each sample, Rietveld analysis of the XRD patterns were performed using GSAS^®^ [19]. For the refinement, the peak shapes were modeled with a pseudo-Voigt distribution and the background with eight Chebyshev polynomials. In each case, a scale factor, four peak shape variables, unit cell and phase parameters, Zero and POLA correction were applied to obtain the structural parameters. Based on the particularities of the variations in the diffraction patterns from both HAp types, the average crystallite size was calculated according to the following expression [20]:

(1) $$\phi =18,000\times 0.9\times \frac{\lambda }{\pi (L_{x}\;+\;ptec)}$$

where the cathode wavelength *λ* = 1.5406 Å [19] and the values for the parameters *L_x_* and *ptec* were obtained from the refinements and were related to the mean diameter of the effective section of the crystallite to the radiation and the crystallite size in the direction of the beam, respectively. The *a*,*b*-plane orientation degree of BHAp and CHAp was calculated with the intensities of the (300), (211) and (002) reflections according to expression:

*Orientation degree of the a,b-plane* = [*I*_300_/(*I*_300_ + *I*_211_ + *I*_002_)] × *100%* (2)

Moreover, the Raman scattering measurement was performed with a micro-Raman spectrometer (LabRam HR-evolution, Horiba, NY, USA) using a He-Ne laser with a wavelength excitation of 632.8 nm, incident power on the sample of 20 mW and spot size 1 µm. All spectra were normalized to the intensity of *v*_1_*PO*_4_^3−^ vibrational mode.

### 2.3. Biocompatibility Assessment

#### 2.3.1. Simulated Body Fluid (SBF) Immersion

All samples were sterilized with UV light for 12 h before in vitro testing. The 4 h sintered set of samples was immersed in SBF previously prepared according to the procedure described by Kokubo [21].

The soaking tests were carried out in closed polyethylene containers with 30 mL of SBF solution, kept at a constant temperature of 36.5 °C in a controlled environment. The chosen immersion times were 24, 72 and 144 h. After the extraction, the specimens were rinsed in distilled water and dried at room temperature. The structural and microstructural characteristics after SBF immersion were measured using XRD and SEM techniques as described above.

#### 2.3.2. Cell Culture and Exposure

Human fetal osteoblastic cells (hFOB 1.19) (American Type Culture Collection Number: CRL-11372) were cultured in Dulbecco’s Modified Eagle Medium (DMEM) with 10% fetal bovine serum and 1% antibiotics. The hFOB 1.19 cells were maintained in a humidified incubator at 37 °C and 5% CO_2_ atmosphere with a constant cell passaging every three days. Before cell culture, all the HAp ceramics were sterilized by UV light for 12 h each side. According to the ISO 10993-5 standard [22], cell metabolic activity (MTS assay) and cell cytotoxicity (LDH assay), were measured by direct contact and extracts tests.

The effect of ceramics surface conditions on the cell viability was evaluated by the direct contact method. The roughness in all the ceramics was homogenized by grinding the surface using a solution of 800 grit SiC powder with ethanol, for 30 min. After sterilization, the specimens were allocated into 24 multi-well plates. The hFOB 1.19 cells (cell density: 5 × 10^3^ cells/cm^2^) were thereafter added into each well to be incubated for 24 h, 72 h and 144 h at 37 °C with 5% CO_2_. The culture medium was renewed every three days.

On the other hand, the extracts method was used to investigate the lixiviation potential and the influence of the ceramics chemical composition on cell viability. The extraction process was performed according to the ISO 10993-12 standard method [23], using DMEM as extraction medium, a ratio of 0.1 g/mL and an exposure time of 24 h. After the exposure time, dilutions with 0%, 12.5%, 25%, 50%, 75%, and 100% of the extract concentration were prepared and located on 96-well plates. The hFOB 1.19 cells (cell density: 5 × 10^3^ cells/cm^2^) were added into each well to be incubated for 72 h.

- Cell mitochondrial activity

MTS (Abcam, Cambridge, UK) -3-(4,5-dimethylthiazol-2-yl)-5-(3-carboxymethoxyphenyl)-2-(4-sulphophenyl)-2*H*-tetrazolium inner salt solution assay was used. After the incubation periods, the culture medium in each well was replaced with a solution of 450 × 10^−3^ mL DMEM and 50 × 10^−3^ mL MTS reagent. Samples were incubated for 4 h at 37 °C and the absorbance was read at 490 nm with a reference wavelength of 690 nm in a multiwell plate reader (Infinite 200, Tecan Group Ltd., Männedorf, Switzerland). The absorbance values were directly proportional to the number of metabolically active cells.

- Cytotoxicity determination

Cell membrane damage was assessed by measuring the lactate dehydrogenase release using LDH assay. The positive controls for cytotoxicity were prepared by adding 5% Triton-X100 (Merck, KGaA, Darmstadt, Germany) and incubating them for 30 min at 37 °C. Supernatant aliquots of 50 × 10^−3^ mL from each well were transferred to a new 96-well plate followed by an addition of 50 × 10^−3^ mL of LDH assay mixture from LDH detection kit (Roche Applied Sciences, Basel, Switzerland). After 30 min of incubation the absorbance was read at 490 nm with a reference wavelength of 690 nm in a multiwell plate reader (Infinite 200, Tecan Group Ltd., Männedorf, Switzerland).

- Statistical analysis

The statistical analysis was performed using the software GraphPad Prism 5^®^ (GraphPad Software, Inc., San Diego, CA, USA). The absorbance values measured in the MTS and LDH assays were analyzed (mean, SD, *n* = 6) using a two-way ANOVA, considering a statistical significance *p* < 0.05.

## 3. Results and Discussion

### 3.1. Microstructure

**Figure 1:**
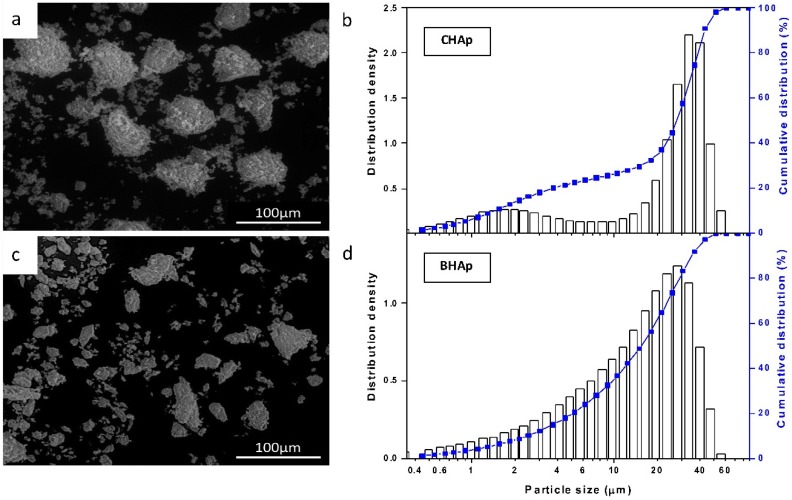
Morphology and particle size distribution for: CHAp (**a**,**b**); and BHAp (**c**,**d**).

The morphological characteristics of the CHAp and BHAp powders are shown in Figure 1a,c, respectively. In Figure 1b,d, the results of particle size distribution measured by laser scattering is also shown in terms of the distribution density and the cumulative size distribution from both type of HAp powders.

As mentioned in the Experimental Section, both powders were processed under the same conditions (sieved to sizes below mesh 200) seeking to have similar particle size distribution. The size distribution in combination with the micrographs show however differences in both size and morphology. For CHAp powder a mixture between spherical agglomerates and fine particles were observed with a bimodal distribution and a maximum frequency at 33 μm (*x*_50_ = 27.63 ± 0.21 μm; *x*_90_ = 56.86 ± 0.79 μm). The size distribution of the fine particles oscillates between 0.5 and 8.22 μm.

In the case of BHAp, rather heterogeneous shapes with a wider size distribution oscillating between 0.5 and 60 μm but in general tending to smaller particle sizes (*x*_50_ = 17.07 ± 0.07 μm; *x*_90_ = 31.82 ± 0.06 μm) was observed. In fact, 90% of the BHAp powder was below 32 μm.

**Figure 2:**
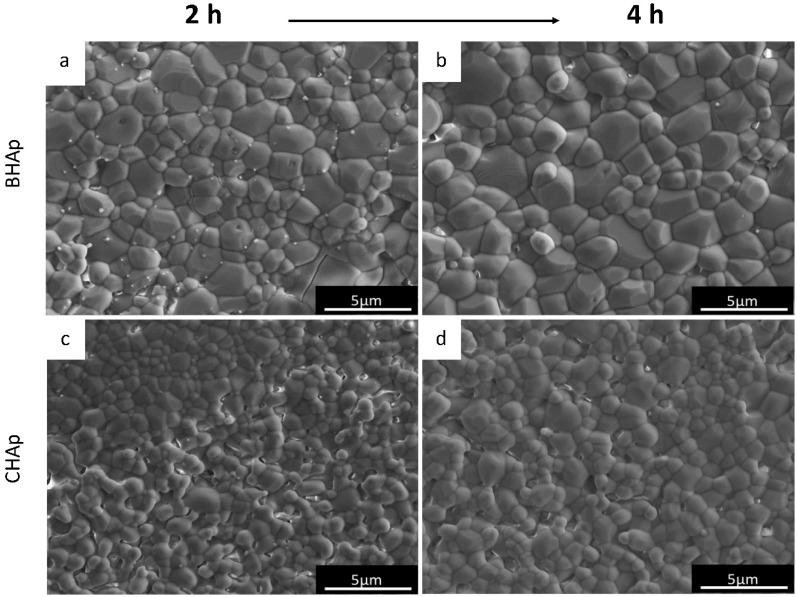
SEM micrographs of sintered ceramics after: 2 h (**a**,**c**); and 4 h (**b**,**d**).

The microstructural details of both HAp ceramics after sintering during 2 h (a, c) and 4 h (b, d) are shown in SEM micrographs from Figure 2. As mentioned before, the processing conditions clearly lead to differences, for instance in the grain size, which is affected by both sintering time (grain coarsening proportional to time) and type of HAp. For the BHAp a grain size of 1.18 ± 0.15 μm was measured after sintering for 2 h and (1.53 ± 0.18 μm) after 4 h increasing by 30%.

On the other hand, the initial grain size in CHAp was 0.72 ± 0.07 μm with an increase of 22% (0.88 ± 0.06 μm) after 4 h sintering, which was, in both cases, clearly lower than for BHAp. Variations in grain size and thus growth behavior from both HAp types can be explained by the differences in chemical composition, particularly due to the presence of specific ions, such as Na^+^, Mg^2+^ and CO_3_^2−^, from the bovine derived bone. Similar behavior has been previously reported by Mostafa et al. [24], in which doping with either 0.51 wt % Na^+^ or 0.3 wt % CO_3_^2−^ leads to a fully sintering stage of HAp at 1100 °C.

The mentioned characteristics are also related to the variation of the porosity and mechanical properties with the sintering time for both HAp, as shown in Table 1. Although the initial porosity of the green samples is almost the same, after sintering a lower porosity was observed for BHAp.

### 3.2. Structural Analysis

#### 3.2.1. Crystallographic Details (XRD)

**Figure 3:**
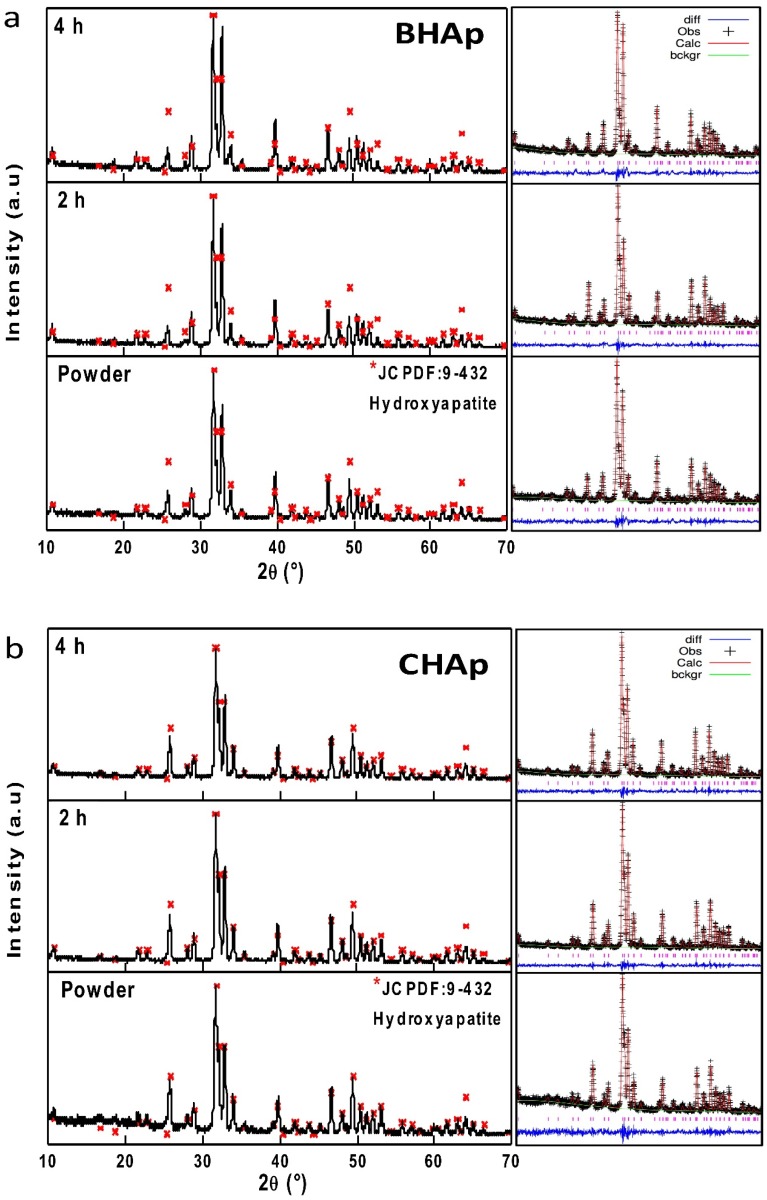
XRD patterns and Rietveld refinements of: BHAp (**a**); and CHAp (**b**) powders and sintered ceramics after 2 h and 4 h. A single phase of HAp (JCPDF 9-432) was identified in BHAp and CHAp.

The results of X-Ray diffraction for the BHAp and CHAp powders and sintered ceramics at 2 h and 4 h are shown in Figure 3a,b, respectively.

In both samples, a single phase of HAp is identified according to the JCPDF 9-432 [10]. However, the influence of sintering time from each type of HAp is only traceable at the level of unit cell characteristics i.e., lattice parameters and crystallite size. As mentioned before, these changes were addressed by Rietveld refinement. In all cases, coefficient of goodness from the refinements (*χ*^2^) is less than 3, which is a value that evidences a correct estimation of the structural parameters, as shown in Table 2. Additionally, the March–Dollase method for preferential orientation was used taking the (002), (211) in the CHAp and (211), (300) for the BHAp planes, which were selected based on the differences between the experimental and theoretical reflection lines.

**Figure 4:**
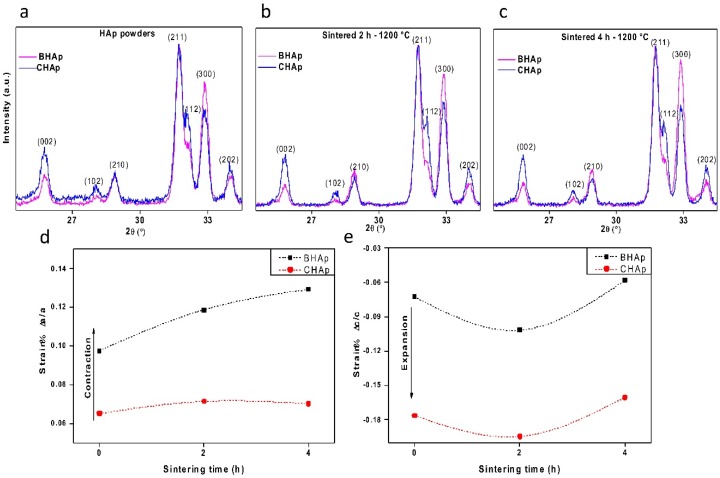
Variations in the XRD peaks intensities in (0 0 2), (2 1 1), (3 0 0) for the bovine-derived HAp and the synthetic Hap: (**a**) powders; and (**b**) 2 h and (**c**) 4 h sintered ceramics. (**d**,**e**) Deformation of lattice parameters in (**a**,**c**).

Figure 4a–c shows the results of variations in the peak intensities for both types of HAp powders as well as for specimens sintered at 2 h and 4 h. The peak with the highest intensity is observed at 31.74° of two theta, which corresponds to the main peak of hexagonal HAp structure. For comparative purposes, all diffraction patterns are normalized taking the (211) plane intensity as a reference. The most significant differences in terms of intensities and peak shifting are observed in the range from 22° to 35° correspondent to the (002), (211) and (300) diffraction planes. From these peaks, a higher intensity in the (002) peak can be clearly observed for the CHAp, whereas for the BHAp the (300) peak is higher. Furthermore, for CHAp, the (211) and (121) crystallographic planes form a doublet, which is typical of stoichiometric and highly crystalline hydroxyapatite. This doublet is clearly lower in intensity in BHAp samples.

Accordingly, the CHAp shows an isotropic orientation in which the *a*,*b*- and *c*-planes are randomly oriented while the behavior of BHAp samples approximates to the bone model due to the remaining texture of hydroxyapatite crystals derived from mammalian cortical bone, thus showing a greater degree of preferred orientation [25,26,27]. Similar effects have been observed by Murugan et al., in terms of (300) diffraction peak shifting and a decrease of *a*-axes after a fluorination treatment of HAp mainly after a so-called high-temperature method [11], but no further analysis related to texturization effects is discussed. On the other hand, Zhuang et al. reported the synthesis of HAp with preferred orientation by precipitation from an aqueous solution to investigate the effect on protein adsorption [28]. In our case, the texturization is definitively due to both the source of HAp and the processing route including heat treatment and milling steps to obtain hydroxyapatite.

Using the experimental lattice parameters obtained from Rietveld refinements, the strains are calculated taking into account the theoretical values for *a* = 9.432 Å and *c* = 6.881 Å [10,29]. The calculated strains are shown in Figure 4d, e. Briefly, in BHAp, a contraction is observed for *a*, which tends to increase with the sintering time, while *c* remains similar to the initial value. In CHAp, the *c* parameter increases with respect to the theoretical value and remains constant with the sintering time, while the parameter *a* is constant irrespective of sintering treatments.

The crystallite size was additionally calculated with the refinement procedure leading to values of 190 and 143 nm for BHAp and 196 and 211 nm for CHAp after 2 h and 4 h sintering, respectively. The reason for these differences in crystallite size and unit cell volume (Table 2) is undoubtedly due to ionic substitutions in the BHAp since the Na^+^ and Mg^2+^ contents measured by ICP-OES were 0.97 and 0.52 at %, respectively. Na^+^ and Mg^2+^ have a smaller ionic radius (0.95 and 0.65 Å) than Ca^2+^ (0.99 Å) leading to well known lattice deformations in solid solution conditions. An opposite behavior is observed in the CHAp powder as a result of the normal growth of the crystallite size with sintering time. No traces of Na^+^ and Mg^2+^ were found in CHAp samples.

Furthermore, the *Ca*/*P* ratio was also obtained by the refinement procedure based on average multiplicity and occupancy values (*O*) of the *Ca*1 (columnar calcium ions), *Ca*2 (*Ca* forming triangles around *c*-axis) and *P* atoms in the hexagonal unit cell of hydroxyapatite according to the next relationship [9,30,31]

(3) CaP=(4×OCa1)+ (6×OCa2)(6×OP)

Therefore, if the fraction of the atom inside the unit cell changes due to its substitution with other atoms with different ionic radius, the ratio between the atoms in the cell changes, inducing strains in the unit cell and hence variations in the lattice parameters. The *Ca*/*P* ratios from both HAp powders obtained by ICP-OES, a well-accepted quantitative method, leads to values of 1.63 and 1.57 for CHAp and BHAp, respectively. In comparison, the calculated values from Equation (3) show ratios of 1.66 and 1.60 for the CHAp and BHAp respectively, which corresponds to a difference of ~3% between both methods, thus confirming the validity of the calculated values obtained from the Rietveld refinement of XRD patterns. 

Finally, contents of heavy elements measured by ICP-OES are shown in Table 3. The values obtained for both HAp are within acceptable limits defined by ISO 13779-1 [32].

#### 3.2.2. Raman Spectroscopy

**Figure 5:**
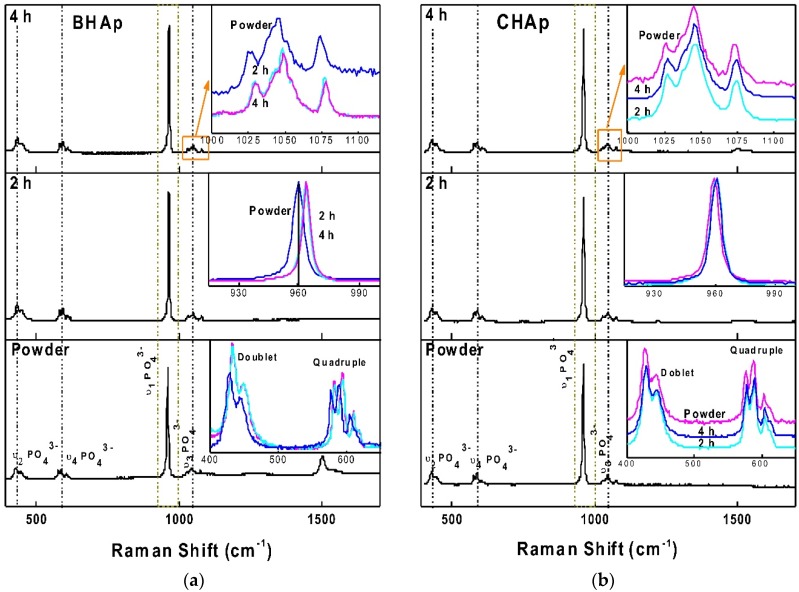
Raman spectra with the main bands for (**a**) BHAp and (**b**) CHAp, showing the position of vibration modes for powders and HAp sintered ceramics at 2 h and 4 h.

The Raman spectra for BHAp and CHAp sintered for 2 h and 4 h are shown in Figure 5. For both spectra, the *v*_1_PO_4_^3−^ band close to 960 cm^−1^, which is the main characteristic for HAp and a double band between 400–450 cm^−1^ assigned to the *v*_2_PO_4_^3−^ HAp band are identified [29,30]. For the *v*_1_PO_4_^3−^ vibrational mode in BHAp, after sintering process, a wave number shift of ~4 cm^−1^ as a possible result of crystalline ordering [33].

The *v*_4_PO_4_^3−^ domain appears between 550 and 650 cm^−1^ and is identified as a triple band in BHAp samples assigned to carbonated apatite with a slight shoulder at 613 cm^−1^, in comparison with a quadruple band detected for CHAp assigned to stoichiometric HAp [33]. Within 1000–1100 cm^−1^, seven vibration modes are detected for CHAp attributed to *v*_3_PO_4_^3−^ band for stoichiometric HAp while only three bands, typical for B-type carbonated apatite, are observed in BHAp samples for the same region [33,34].

Additionally, for BHAp powder, four bands were detected between 1500 and 1935 cm^−1^ corresponding to residual CaO [35], after sintering process (2 h and 4 h) these bands completely vanished. A summary of the identified bands and its assignations for both HAp powders, according to [33,34,35,36] are tabulated in Table 4.

### 3.3. In Vitro Assessment in SBF

**Figure 6:**
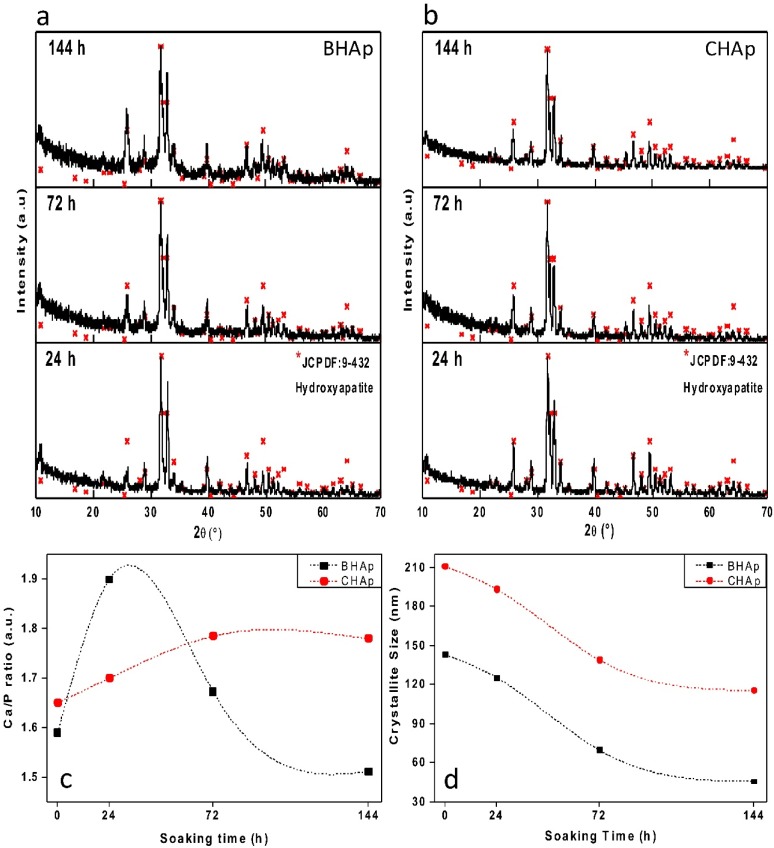
XRD patterns for bovine-derived (**a**) and synthetic (**b**) HAp sintered ceramics at 1200 °C for 4 h after 24 h, 72 h and 144 h of exposure to simulated body fluid. Changes in *Ca*/*P* ratio (**c**) and crystallite size (**d**) obtained by Rietveld refinements.

The XRD patterns of BHAp and CHAp ceramics sintered at 1200 °C for 4 h, after 24 h, 72 h, and 144 h of exposure in SBF are presented in Figure 6a,b. Comparing with the original XRD pattern, an increase in the mean width of the peaks and a decrease in their relative intensity is observed. For the BHAp samples, the (012) plane vanishes after 72 h of immersion whereas the intensity of the (002) plane increases with the immersion time. On the other hand, for the CHAp samples a peak broadening is not noticeable and the original reflection planes are preserved throughout the immersion time. For each immersion step, the crystallite size and *Ca*/*P* ratio are calculated using Equations (1) and (3), as shown in Figure 6c,d, respectively. In the *Ca*/*P* ratio, two different trends are observed: (a) for the BHAp, an increasing tendency due to the *Ca*-rich layer formation, followed by a decrease generated by a *Ca*-poor surface due to the interaction with the fluid [37]; and (b) for CHAp, a monotonic increasing tendency is observed.

In Figure 6d, the decrease in crystallite sizes for both HAp types is shown, which is associated to an amorphous state of the surface due to the formation of the bone-like apatite layer. Larger changes are however observed for the BHAp, which can be associated with the degree of orientation in the *a*,*b-*plane, due to the exposure of OH^−^ channels of the *c* axis, generating a surface with higher electronegativity, increasing their reactivity when exposed to body fluids [9,28,38].

**Figure 7:**
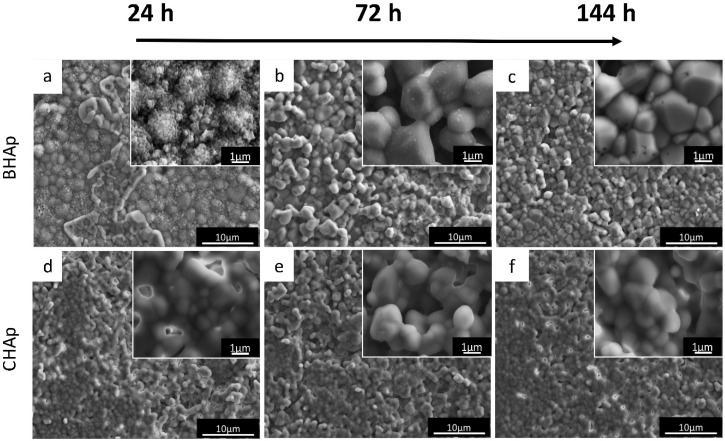
Microstructural characterization by SEM micrographs for BHAp and CHAp sintered ceramics at 1200 °C for 4 h, showing changes in morphology after: (**a**,**d**) 24 h; (**b**,**e**) 72 h; and (**c**,**f**) 144 h of exposure to simulated body fluid.

The microstructural changes of the selected BHAp and CHAp ceramics after immersion for 24 h (Figure 7a,d), 72 h (Figure 7b,e), and 144 h (Figure 7c,f) in SBF are shown in Figure 7. Both grain size and texturization change as a function of immersion time for BHAp are observed. Specifically, for the BHAp samples, the grain size goes from 1.90 ± 0.19 μm at 24 h to 1.47 ± 0.18 μm after 144 h of exposure, in contrast with the CHAp samples where the grain size remains constant at around 1 μm even after 144 h of exposure. These changes could be related to the incorporation of Na^+^, Mg^2+^, Ca^2+^, K^+^ ions in the HAp structure as a result of the interaction with the physiological environment and serve as a proof to the onset in the formation of a new phase, that stabilizes with the exposure time. According to Kim et al., the phase formed is consequence of the ion exchange and corresponds to bone-like apatite [33]. The first stages of formation involve the precipitation of calcium ions on the HAp, generating an amorphous scale of calcium phosphate, which over time crystallizes in bone-like apatite. This process can be described as a dissolution/reprecipitation reaction process, which takes place faster for carbonated than for stoichiometric apatite [39].

### 3.4. In Vitro Assessment in Cell Culture

To investigate the innocuity of HAp materials, as mentioned before different cell viability assays in an osteoblast cell line were performed. Cellular response analysis in implanted materials encompass chemical and biological processes, related with surface characteristics, element ionization and protein adsorption, and should lack cell toxicity.

**Figure 8:**
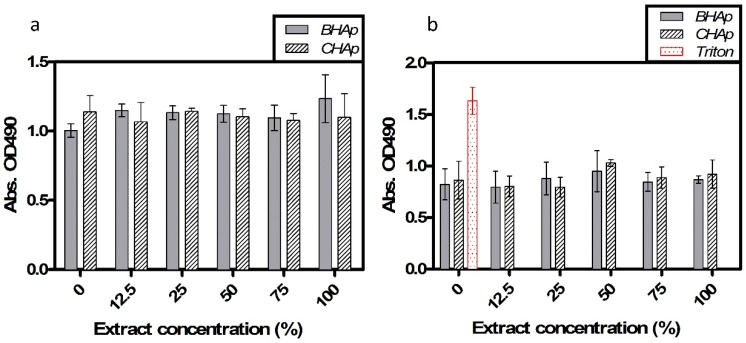
Cell viability after 72 h of exposure to different extract concentrations: (**a**) MTS assay; and (**b**) cytotoxicity by LDH assay. Data were normalized and compared (mean, SD, *n* = 6) using a two-way ANOVA, no significant differences considering *p* < 0.05.

Cell viability results after 72 h of exposure to different extract concentrations are shown in Figure 8. Cell metabolic activity and membrane damage, used as indicators of cell viability, were evaluated after exposure, showing that in all the studied conditions the absorbance, related to cell metabolic activity (MTS) and cell death (LDH) is constant after each extract concentration.

The statistical analysis showed no differences in the cell behavior after the exposure to different extract concentrations. Similarly, the two-way ANOVA shows that HAp type is not an influential factor for cell viability or cytotoxicity. Thus, both types of HAp clearly show a non-toxic cell behavior related to the lixiviation potential and ceramics chemical composition.

**Figure 9:**
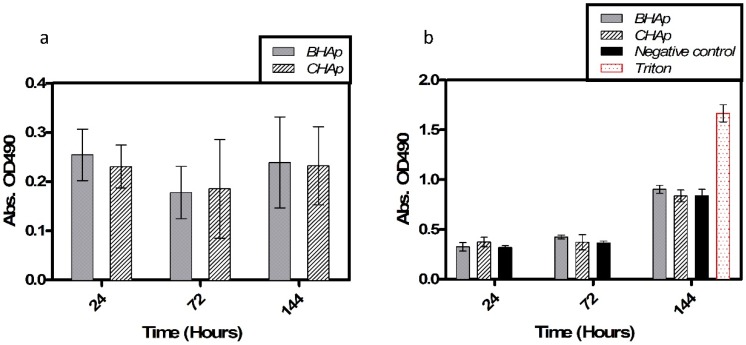
Cell viability of hFOB cells after 24 h, 72 h and 144 h of exposure to samples: (**a**) MTS assay; and (**b**) cytotoxicity by LDH assay. Data were normalized and compared (mean, SD, *n* = 6) using a two-way ANOVA, no significant differences considering *p* < 0.05.

In addition, Figure 9a shows the metabolic activity during the different periods of exposure by direct contact of the cells with the BHAp or CHAp, as indicative of cell viability, proliferation and possibly cell integration and biocompatibility, using the MTS assay. No statistically significant differences between the BHAp and CHAp are observed. Additionally, there was no increase in the absorbance with the time of exposure, which seems to indicate a low capacity of the cells to proliferate. Although no cell toxicity is evident with BHAp, an increased cell adhesion and integration represented by proliferation was not evident, possibly due to the endpoint tested. Further studies testing more specific endpoints of cell adherence, proliferation and cell differentiation to confirm bone regeneration (i.e., alkaline phosphate and osteocalcin, amongst others) are underway. On the other hand, LDH measurements, shown in Figure 9b, indicate that no significant differences in the induced cytotoxicity between the BHAp and CHAp are present.

## 4. Conclusions

- The in-house developed processing route allowed obtaining highly crystalline HAp from bovine bone. The Na^+^ and Mg^2+^ ions intrinsically presented in BHAp seem to influence the sintering behavior evolving to ceramics with lower porosity and coarser microstructure compared to those obtained with synthetic HAp.
- At the structural level, the main differences between the BHAp and CHAp consist in an increase in the orientation degree of *a*,*b*-plane and the contraction of *a* lattice parameter of HAp unit cell. These differences are related to the BHAp source which may have a positive effect in the in vitro performance, quantifiable at longer immersion periods.
- Despite the clear structural differences between BHAp and CHAp at the unit cell level, the hydroxyapatite obtained from bovine bones has comparable in vitro behavior with the commercial one. This result is very significant considering the positive cost/benefit ratio from BHAp and thus can be used with no restrictions for biomedical applications in the same way as CHAp.

## Figures and Tables

**Table 1:** Properties of the HAp ceramics from different sources sintered at 1200 °C for 2 h and 4 h.

Sample (Sintered at 1200 °C)	Open Porosity (%)	Vickers Hardness (HV)	Grain Size (µm)
BHAp green sample	35.04 ± 0.08	-----	-----
BHAp 2 h	16.06 ± 0.25	227.78 ± 28.80	1.18 ± 0.14
BHAp 4 h	12.09 ± 1.12	332.30 ± 77.40	1.53 ± 0.18
CHAp green sample	35.11 ± 0.23	----	-----
CHAp 2 h	30.40 ± 2.85	80.21 ± 10.46	0.72 ± 0.06
CHAp 4 h	27.17 ± 0.39	109.40 ± 19.70	0.89 ± 0.06

**Table 2:** Structural parameters obtained from XRD patterns analyzed by Rietveld refinement.

Source	Sample	a (Å)	c (Å)	Volume (Å^3^)	*Ca/P* Rietveld	*Ca/P* ICP	*χ*^2^
Theoretical		9.432	6.881	528.8	1.67	-	-
BHAp	Powder	9.423	6.886	529.8	1.60	1.57	2.61
	2h	9.421	6.891	529.6	1.63	---	1.75
	4h	9.420	6.885	529.1	1.59	---	2.15
CHAp	Powder	9.426	6.893	530.4	1.66	1.63	2.25
	2h	9.425	6.894	530.4	1.69	---	2.45
	4h	9.425	6.892	530.2	1.65	---	2.36

**Table 3:** Heavy elements content of BHAp and CHAp samples measured by ICP-OES.

Element	BHAp (ppm)	CHAp (ppm)	Values accepted ISO 13779-1:2008 (ppm)
As	0.00	0.09	<3
Cd	0.00	0.00	<5
Pb	0.02	0.03	<30
Hg	0.00	0.00	<5

**Table 4:** Comparison of Raman spectroscopic band assignments for stoichiometric HAp, carbonated HAp type A and B, bone and experimental values from BHAp and CHAp.

Assignment	Reported [33,34,35,36]				Experimental Values (Powders)		
	Stoichiometric HAp	CAp Type A	Cap Type B	Bone	CaO	BHAp	CHAp
*v*_1_PO_4_^3^^−^	964	947	961	961		960	960
		957					
*v*_2_PO_4_^3−^	433	440	432	432		429	427
	448		445	452		444	442
*v*_3_PO_4_^3−^	1029	1018	1026	1032		1026	1026
	1034						1033
	1041						1038
	1048	1031	1047	1044		1045	1045
	1057						1053
	1064						1064
	1077	1059	1070	1071		1072	1074
*v*_4_PO_4_^3−^>	580	579	579	584		579	577
	591	589	590	590		589	589
	607	608	609	611		605	604
	614						613
Type A *v*_1_CO_3_^2−^		1107		1103		1109	
OH stretch	3573		3576	NO		3571	3570
CaO					1500	1500	
					1550	1545	
					1772	1771	
					1935	1930	
